# Low Doses of Deoxynivalenol and Zearalenone Alone or in Combination with a Mycotoxin Binder Affect ABCB1 mRNA and ABCC2 mRNA Expression in the Intestines of Pigs

**DOI:** 10.3390/toxics12040297

**Published:** 2024-04-17

**Authors:** Nikolay Nikolov, Tsvetelina Petkova, Rumen Binev, Aneliya Milanova

**Affiliations:** 1 Department of Internal Non-Infectious Diseases, Faculty of Veterinary Medicine, Trakia University, 6015 Stara Zagora, Bulgaria; nikolay.nikolov@trakia-uni.bg (N.N.); rumen.binev@trakia-uni.bg (R.B.); 2 Department of Pharmacology, Animal Physiology, Biochemistry and Chemistry, Faculty of Veterinary Medicine, Trakia University, 6015 Stara Zagora, Bulgaria; tsvetelina.petkova@trakia-uni.bg

**Keywords:** ABC efflux transporters, deoxynivalenol, zearalenone, pig

## Abstract

Mycotoxin binders, in combination with enzymes degrading some mycotoxins, contribute to feed detoxification. Their use reduces economic losses and the negative impacts of mycotoxins on animal health and productivity in farm animals. The aim of this study was to evaluate the efficacy of a mycotoxin detoxifier on the expression of the ATP-binding cassette efflux transporters ABCB1 mRNA and ABCC2 mRNA, which transport xenobiotics and thus have a barrier function, in the tissues of pigs exposed to low doses of deoxynivalenol (DON, 1 mg/kg feed) and zearalenone (ZEN, 0.4 mg/kg feed) for 37 days. The levels of expression were determined by an RT-PCR, and the effect of the mycotoxin detoxifier (Mycofix Plus3.E) was evaluated by a comparison of results between healthy pigs (*n* = 6), animals treated with DON and ZEN (*n* = 6), and a group that received both mycotoxins and the detoxifier (*n* = 6). A significant downregulation of ABCB1 mRNA and ABCC2 mRNA was observed in the jejunum (*p* < 0.05). A tendencies toward the downregulation of ABCB1 mRNA and ABCC2 mRNA were found in the ileum and duodenum, respectively. The mycotoxin detoxifier restored the expression of ABCB1 mRNA to the level found in healthy animals but did not restore that of ABCC2 mRNA to the level of healthy animals in the jejunum.

## 1. Introduction

Animal feed is commonly infected with more than one fungal species which can pose significant risks to both animal and human health. Fusarium fungi such as *Fusarium culmorum*, *Fusarium graminearum*, and *Fusarium cerealis* can produce multiple mycotoxins. Zearalenone (ZEN) and Deoxynivalenol (DON) are products of these fungal species, which explains why they are often found together in feed [1,2]. These two mycotoxins are relatively stable and can persist under various environmental conditions [3]. ZEN is classified as a non-steroidal estrogenic mycotoxin due to its ability to exert effects similar to those of estrogens [4,5]. Its toxicity has been associated with reduced fertility and litter size, uterine enlargement, changes in serum levels of progesterone and estradiol in laboratory animals, as well as alterations in fertility and reproduction in pigs [6,7,8,9]. Pigs are particularly sensitive to ZEN but also highly sensitive to DON [10,11,12]. Low-dose exposure to DON affects the intestinal barrier and the intestinal immune system, which may significantly impact pig health and performance [13,14,15,16]. DON can negatively affect the morphology of the small intestine in pigs by reducing villus height in the jejunum, thereby compromising nutrient absorption and digestion [13,17]. The highly toxic DON is rapidly absorbed from contaminated feed in the gastrointestinal tract, with the highest bioavailability of 85% observed in pigs [18,19]. This rapid absorption and wide distribution to various tissues contribute to its toxic effects on multiple organs and systems [20]. It is recognized that the effects of a single mycotoxin can vary significantly when compared to the effects observed after co-exposure to a mix of mycotoxins. Thapa et al. [21] reported controversial data from in vitro and in vivo studies ranging from synergistic to antagonistic toxic effects after co-exposure to DON and ZEN. Combined treatment with DON and ZEN resulted in a higher reduction in cell viability in HepG2 cells [22]. Toxicokinetic studies demonstrated that exposure to low doses of DON and ZEN led to the accumulation of DON in the tissues of healthy gilts without altering the disposition of ZEN [23]. Jia et al. [24] observed that piglets co-exposed to both toxins showed significantly decreased body weight gains and average daily feed intakes, indicating a synergistic negative impact on intestinal function and subsequent systemic inflammation. The absorption of mycotoxins through the intestinal epithelium is limited, and their elimination from the body is facilitated by active efflux through ABC transporter proteins [25]. ABC efflux transporters are part of the first line of defense in the intestines, and their function contributes to the elimination of many endogenous substances and xenobiotics, thus protecting tissues [26,27,28]. DON has been described as a substrate for efflux transporters which can also downregulate the expression of ABCB1 mRNA, the gene encoding P-glycoprotein, in the jejuna of pigs [9,15]. Exposure to ZEN at an early stage of life led to the downregulation of ABCB1a and ABCB1b mRNA in the testes of rats [29]. Published data on the effects of DON and ZEN on the expression of ABC efflux transporters are limited to the above-mentioned tissues. The impacts of both mycotoxins on ABCB1 mRNA and ABCC2 mRNA in other tissues are unclear, and it is unknown whether they are limited only to the gastrointestinal tract. According to our knowledge, the effect of co-exposure to both mycotoxins on the investigated transporters has not been described in the scientific literature.

These findings underscore the complexity of the interactions between DON and ZEN and their effects on biological systems. Further research is needed to understand the mechanism of action of the DON and ZEN combination in order to develop an optimal strategy for reducing their toxicity. Mycotoxin detoxification agents have been utilized for decades to reduce absorption and mitigate the adverse effects of mycotoxins on animal health [30,31]. However, it was later suggested that simultaneous exposure to low doses of mycotoxins (40 μg/kg b.w. of ZEN and 12 μg/kg b.w. of DON daily with feed) may be necessary for the development of nutritional tolerance to these harmful compounds, leading to questions about the need to administer mycotoxin detoxifiers in young pigs [23]. To date, a highly successful strategy for significant reductions in the adverse effects of mycotoxins present in feed has not been proposed, necessitating further investigations and the acquisition of more data regarding the efficacy of mycotoxin detoxifiers [32]. Little is known about the protective effect of these compounds on the expression and function of ABC efflux transporters encoded by the ABCB1 and ABCC2 genes.

The aim of the current study was to investigate the effects of exposure to a combination of deoxynivalenol (DON) and zearalenone (ZEN), administered at low doses to pigs through their feed, on the expression levels of ABCB1 mRNA and ABCC2 mRNA. Additionally, the impact of the combination of a mycotoxin binder based on bentonite, enzymes, and phytogenic substances, DON and ZN, on the expression of ABCB1 and ABCC2 mRNAs was evaluated.

## 2. Materials and Methods

### 2.1. Animals

The experiments were carried out after obtaining ethical approval from the Bulgarian Food Safety Agency (License 271/2 June 2020). The zoo hygiene requirements of the premises where the experimental animals were housed and where the experiment was conducted corresponded to Ordinance No. 20/1 November 2012, which outlines the minimum requirements for the protection and humane treatment of experimental animals, as well as requirements for facilities for their use, breeding, and/or delivery. Additionally, Ordinance No. 21/14 December 2005, which specifies the minimum requirements for protection and humane treatment in pig farming, was considered in the current experiment.

Eighteen one-month-old piglets from both sexes, hybrids of ♀ Great White × English Landrace and ♂ Pietren, were obtained from the pig farm “Mihaela—V. Zhelyazkova” (Khan Asparuhovo, Stara Zagora, Bulgaria). The average body weight of the pigs at the beginning of the experiment was 8.99 ± 1.02 kg. The animals were accommodated in a Biobase at the Faculty of Veterinary Medicine, Trakia University. The ambient temperature was 24 °C at the beginning of the experiment and 18 °C at the end, with a relative humidity of about 70%. Air exchange was carried out through natural ventilation or through a ventilation system, maintaining an air movement speed of 0.2–0.5 m/s. The experimental animals were provided with a free floor area of 0.4 m^2^ per pig. The light cycle consisted of 12 h of daylight and 12 h of darkness. The control and experimental groups were located in different sections in the same room. Access to feed and water was supplied ad libitum through automatic bunker feeders for the respective animal species and through a nipple system for the water.

### 2.2. Study Design

After a 5-day acclimatization period, the pigs were randomly assigned to three groups, *n* = 6 each. The first group included healthy pigs (*n* = 6) which served as controls. They were fed balanced feed according to their age (a starter produced by Melhran EOOD, Stara Zagora, Bulgaria). The pigs from the second group received the same feed supplemented with DON at a dose of 1 mg/kg feed and ZEN at a dose of 0.4 mg/kg feed (Fermentek Ltd., Jerusalem, Israel). DON and ZEN were added to the commercial pig diet as purified toxins. They were selected based on a literature review and in order to keep the experiment close to field conditions [12,15]. The third group of pigs was fed the same feed as the second group, with the addition of bentonite as Mycofix Plus3.E at a dose of 2 kg/ton feed (Biomin, Inzersdorf-Getzersdorf, Austria). It contained the following components: a synergistic blend of minerals, biological constituents, a synergistic blend of minerals, a biological constituent, BBSH 797, phytogenic substances, and phycophytic constituents. The animals were exposed to mycotoxins and to the combination of DON + ZEN with the mycotoxin binder for 37 days.

At the end of the experiment, the pigs were humanely euthanized according to Appendix No. 6, Art. 28, para. 4 of Regulation No. 20/1 November 2012, using a high dose of anesthetic—Euthanasin “N” (Vetprom, Radomir, Bulgaria)—at a dose of 20 mL/pig intravenously in the v. auricularis lateralis after preliminary sedation with Anaket (Richter Pharma AG, Wels, Austria) at a dose rate of 20 mg/kg b.w. Thereafter, tissue samples from the liver, kidney, duodenum, jejunum, ileum, adrenal gland, ovaries, and testes were immediately snap-frozen in liquid nitrogen. All samples were stored at −80 °C until their analysis.

### 2.3. RNA Isolation and cDNA Synthesis

As a first step in the RT-qPCR analysis, total RNA was isolated from the tissues using Trizol (TRI Reagent, Sigma Aldrich, St. Louis, MO, USA) according to the manufacturer’s instructions. The concentrations and purity of the extracted total RNA were determined spectrophotometrically at 260/280 nm (Agilent Cary 60 UV-Vis Spectrophotometer, Agilent Technologies, Santa Clara, CA, USA). Samples were stored at −80 °C for less than seven days until reverse transcription. Reverse transcription was performed using a Revert Aid First Strand cDNA synthesis kit (Thermo Fisher Scientific, Waltham, MA, USA) following the manufacturer’s protocol. cDNAs were synthesized from 5 µg of total RNA from each sample. The reaction mixture was incubated for 60 min at 42 °C, the enzyme was heat-inactivated at 70 °C for 5 min, and the samples were rapidly cooled to 4 °C. The reverse transcription was conducted using RT-PCR equipment, a Gentier 96E/96R (Xian Tianlong Science and Technology, Xi’an, China). The cDNA was stored at −80 °C.

After reverse transcription, an RT-PCR was conducted using specific primer pairs for the genes of interest, ABCB1 and ABCC2, as well as for the reference genes GAPDH and HPRT (Table 1). The primers were obtained from Sigma Aldrich (Merck, Darmstadt, Germany). The final reaction volume was 20 µL, consisting of 5 µL of diluted cDNA mixed with 10 µL 2× QuantiNova SYBR Green PCR Master Mix (QIAGEN, Venlo, The Netherlands), 1 µL of each forward and reverse primer, and 3 µL sterile RNase-free water. The primers were used at a concentration of 10 pmol/µL. The amplification was performed on RT-PCR equipment, using a Gentier 96E/96R. The initial hot start was carried out for 2 min at 95 °C, followed by 40 cycles of denaturation for 5 s at 95 °C and combined annealing/extension for 30 s at 60 °C. Negative controls were included for each primer pair, comprising all of the reagents for the RT-PCR mix without the addition of cDNA. All samples were analyzed in duplicate. A melting curve analysis confirmed the specificity of the PCR products. Efficiencies for each reaction were estimated using LinRegPCR version 7.0 software. The data from PCR reactions were analyzed according to the method described by Vandesompele et al. [33]. HPRT and GAPDH were used as reference genes for the normalization of the expression levels of the genes of interest. The criteria for selecting reference genes were a lack of treatment effect and expression variation.

### 2.4. Statistical Analysis

The results are presented as arithmetic mean and standard deviation values. The expression levels of the genes of interest in each tissue were analyzed, and comparisons between the control group and the groups that received different treatments were performed using the Mann–Whitney test (Statistica for Windows 10, StatSoft, Inc., Tulsa, OK, USA). Differences were considered statistically significant at *p* < 0.05.

## 3. Results

The investigated ABC efflux transporter proteins were determined at the mRNA level in all studied tissues. Although the animals were of the same age and breed, significant interindividual differences were observed in the expression levels of ABCB1 mRNA in the adrenal gland and ABCC2 mRNA in the kidney and duodenum.

**Figure 1:**
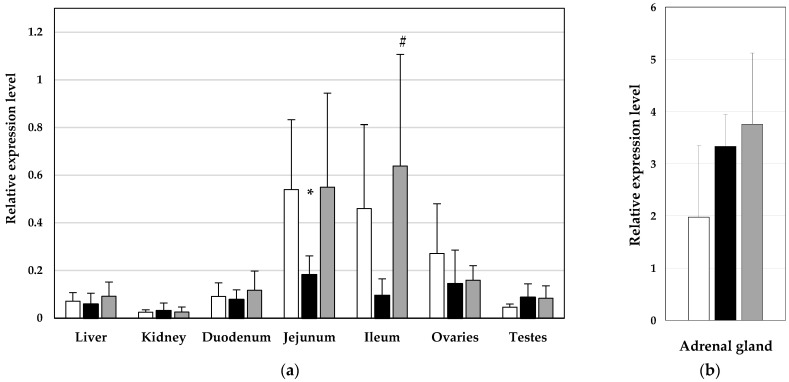
(**a**) Relative expression levels of ABCB1 mRNA in liver, kidney, duodenum, jejunum ileum, ovaries, and testes and (**b**) in adrenal gland in healthy pigs (white bars); DON-plus-ZEN-treated pigs (black bars); pigs treated with DON plus ZEN plus mycotoxin binder (Mycofix, gray bars). Expression was normalized against Hypoxanthine-guanine phosphoribosyl transferase (HPRT) and glyceraldehyde3-phosphate dehydrogenase (GAPDH). * statistically significant differences between controls and DON-plus-ZEN-treated animals at *p* < 0.05; # statistically significant differences between pigs treated with DON plus ZEN and animals treated with DON plus ZEN plus Mycofix at *p* < 0.05.

The relative expression levels of ABCB1 mRNA in the tissues of pigs from all three experimental groups are presented in Figure 1. High levels of mRNA of this gene of interest were found in the adrenal gland, jejunum, and ileum of healthy, growing pigs. Exposure to DON plus ZEN led to a statistically significant downregulation of ABCB1 mRNA in the jejunum (*p* < 0.05) and a tendency toward a decreased level of expression of the studied gene in the ileum. The co-administration of an adsorbent containing bentonite restored ABCB1 mRNA levels to those detected in healthy animals. A tendency toward an increase in ABCB1 mRNA expression in the adrenal gland was observed in both groups treated with DON plus ZEN, with and without the adsorbent, compared to healthy pigs.

**Figure 2:**
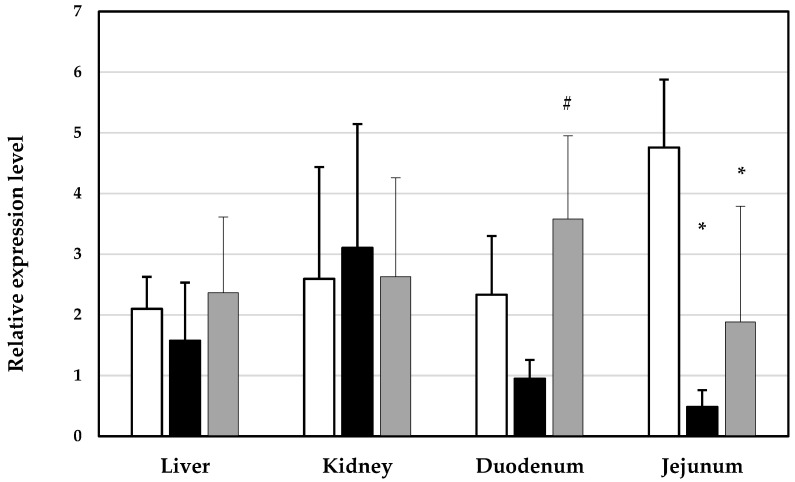
Relative expression levels of ABCC2 mRNA in healthy pigs (white bars); DON-plus-ZEN-treated pigs (black bars); and pigs treated with DON plus ZEN plus mycotoxin binder (Mycofix, gray bars). The expression was normalized against Hypoxanthine-guanine phosphoribosyl transferase (HPRT) and glyceraldehyde3-phosphate dehydrogenase (GAPDH). * statistically significant differences between controls and DON-plus-ZEN-treated animals, with or without Mycofix, at *p* < 0.05; #—statistically significant differences between groups treated with DON plus ZEN and DON-plus-ZEN-plus-Mycofix-treated animals at *p* < 0.05.

The relative expression levels of ABCC2 mRNA are presented in Figure 2. There were no statistically significant differences in the expression levels of this gene of interest in the liver and kidneys among the three groups. However, a tendency toward the downregulation of ABCC2 mRNA was found in the duodena of pigs exposed to the combination of mycotoxins, while it was significantly (*p* < 0.05) changed in the jejunum compared to healthy animals (Figure 2). Despite the tendency toward the restoration of ABCC2 mRNA expression in the jejunum in the group treated with DON plus ZEN plus bentonite (Mycofix) (*p* < 0.05), the average level remained significantly lower compared to that of healthy pigs. ABCC2 mRNA expression in the duodenum was restored to a level similar to that in healthy pigs after the addition of Mycofix. It was significantly lower in the duodena of the group exposed to DON plus ZEN compared to the group treated with DON plus ZEN and Mycofix.

## 4. Discussion

The mycotoxin contamination of feed remains a significant concern for feed safety, potentially leading to various health issues in animals such as reduced growth, impaired immune function, and reproductive problems [35]. One method commonly employed to mitigate the harmful effects of mycotoxins and to reduce the exposure of humans via the food chain is the use of mycotoxin detoxification agents [30,31]. Therefore, this investigation aims to identify the effects of co-exposure to DON and ZEN and the addition of a mycotoxin detoxifier on the expression levels of ABCB1 mRNA and ABCC2 mRNA, two genes encoding efflux proteins that are part of the biological barriers in different tissues, in pigs.

P-glycoprotein, a product of the ABCB1 gene, and a multidrug resistance protein encoded by ABCC2 mRNA, have significant roles in efflux of many xenobiotics, including toxins, decreasing their absorption and distribution or contributing to the faster elimination of these compounds from the body [36,37]. In vitro investigations with Caco-2 cells proved that DON is a substrate of both P-glycoprotein and MRP2 [38,39]. The stomach and the proximal small intestine primarily contribute to the absorption of DON [40]. ABCB1 mRNA was found in the investigated pig tissues and showed high levels of expression in the jejunum, ileum, and adrenal glands, as described earlier [34]. According to our data, it can be inferred that when combined with ZEN, this mycotoxin significantly inhibited the expression of ABCB1 mRNA in the jejunum. A downregulation of this gene was observed by Alizadeh et al. [15] after the short-term treatment of pigs with low doses of DON. These changes are consistent with the morphological alterations observed in jejunal villus height reported by Bracarense et al. [13] and Wu et al. [17]. Furthermore, the observed tendency toward downregulation in the ileum is an additional explanation for the smaller role of these efflux transporters in the formation of the gastro-intestinal barrier in pigs simultaneously exposed to DON and ZEN. The positive adsorbent’s effect in mitigating the impact of DON plus ZEN on the expression of ABCB1 mRNA in the gastrointestinal tract can lead to the recovery of expression levels to those observed in healthy pigs. Data from the literature affirmed that ZEN was not able to affect the mRNA expression levels of ABCB1 in the ovaries and livers of pregnant Wistar rats, which is consistent with the findings of the current experiment with juvenile female pigs [9]. Similarly, there were no changes in ABCB1 mRNA levels in the testes of pigs treated with DON plus ZEN. In contrast, ABCB1a mRNA was upregulated in the SerW3 Sertoli rat cell line after treatment with concentrations of > 5 nM of ZEN, and it was downregulated in the testes of male Sprague Dawley rats [29]. Based on our observations, it might be concluded that low doses of DON plus ZEN did not affect ABCB1 mRNA in reproductive organs such as ovaries and testes in growing immature pigs, which may protect these tissues from toxic compounds. It can be suggested that the variations in these observations depend on the animal species. A tendency toward the upregulation of ABCB1 mRNA in the adrenal gland, a tissue characterized by a high abundance of this gene, could be explained by the role of P-glycoprotein in the secretion of steroid hormones synthetized in the glands [41]. The addition of the mycotoxin binder did not change the observed tendency toward the upregulation of ABCB1 mRNA in this tissue. Although the observed changes in the adrenal gland were statistically insignificant, it can be speculated that they are related to DON exposure and stress, which have been discussed as main causes of necrosis syndrome in swine [42]. It should be mentioned that ZEN and DON significantly changed the expression of genes related to steroidogenesis [43,44]. While ZEN leads to a decrease in the levels of steroid sex hormones, DON stimulates the release of estradiol and testosterone [43,44]. However, the impact of both mycotoxins on the secretion of other adrenal gland hormones is unclear.

ABCC2 mRNA encodes MRP2, an efflux transporter protein involved in the excretion of low-molecular-weight compounds such as GSH, glucuronate and sulfate conjugates, including bilirubin glucuronide, and different drugs and toxins [45]. In vitro investigations with Caco-2 cells demonstrated that ZEN and its reduced metabolites ß-zearalenol and α-zearalenol (α-ZOL) were transported by MRP2 [39]. The lack of changes in the expression of ABCC2 mRNA in the liver and kidneys observed in the current experiment is a prerequisite for the insignificant effect of DON plus ZEN on the elimination of substrates of MRP2 from these tissues. Our findings are similar to the observed lack of changes in ABCC2 mRNA in the livers of pregnant Wistar rats exposed to ZEN [9]. On the contrary, a significant downregulation was observed in the jejunum and a tendency toward decreased expression in the duodenum were noted in the group of pigs treated with DON plus ZEN. These findings are in line with the findings in pregnant Wistar rats treated with ZEN [9]. The duodenum and the jejunum have a major role in controlling the absorption and excretion of nutrients, toxins, and drugs and their metabolites by participating in their secretion through the intestinal wall or limiting their absorption from the intestinal lumen. The significant decrease in the level of ABCC2 mRNA expression in the jejunum as a result of chronic exposure to DON plus ZEN is a prerequisite for the disruption of the barrier functions of the gastrointestinal tract and for drug interactions, which must be evaluated in further functional studies. The administration of the mycotoxin adsorbent was not able to fully restore ABCC2 mRNA expression in the jejunum to the level found in healthy pigs, despite the observed tendency toward its upregulation.

The experiments performed are a first step in the investigation of the effect of DON and ZEN co-exposure on the expression levels of the studied efflux transporters. Although no negative impact of these mycotoxins was detected, even at the applied low dietary levels, more investigations are required to evaluate the clinical relevance of these changes. The effect of the mycotoxin binder used on the mRNA expression of ABCB1 and ABCC2 cannot be entirely excluded. High inter-individual variations, particularly influenced by the number of animals in each group, must be acknowledged as another limitation affecting the follow-up of changes in the expression of ABCB1 mRNA and ABCC2 mRNA. Another limitation of the study is related to the investigation of both ABC efflux transporters at the mRNA level only. Future studies should focus on functional studies in order to better characterize the biological significance of the observed changes.

## 5. Conclusions

The co-exposure of pigs to DON and ZEN via feed led to the downregulation of ABCB1 mRNA and ABCC2 mRNA in the jejunum. The mycotoxin binder used was able to mitigate the negative impact of DON + ZEN co-exposure to a significant extent, although the levels of ABCC2 mRNA were not restored to those found in healthy animals. However, these findings are only a first step toward a better understanding of the roles of both ABC transporters in protecting the body from the harmful effects of co-exposure to DON and ZEN. Further investigations at functional levels could contribute to revealing the clinical significance of potential interactions between substrates of the studied ABC transporters.

## Figures and Tables

**Table 1:** The nucleotide sequences of the specific primers used for the RT-PCR analysis in *Sus scrofa scrofa*.

Gene	NCBI Accession No.	Forward Primer 5′→3′	Reverse Primer 5′→3′	Nucleotide Location
ABCB1	AY825267	TGGCAGTGGGACAGGTTAGTTC	CACGGTGCTTGAGCTGTC	2155–2270
ABCC2	DQ530510	GTGGCTGTTGAGCGAATAAATGAATAC	TGCTGGGCCAACCGTCTG	798–888
HPRT	NM_001032376	ATCATTATGCCGAGGATTTGGA	CCTCCCATCTCTTTCATCACATCT	84–183
GAPDH	AF017079	GGCAAATTCCACGGCACAGTCA	CTGGCTCCTGGAAGATGGTGAT	495–576

ABCB1, ATP Binding Cassette Subfamily B Member 1; ABCC2, ATP Binding Cassette Subfamily C Member 2; HPRT, Hypoxanthine-guanine phosphoribosyl transferase; GAPDH, glyceraldehyde3-phosphate dehydrogenase; NCBI, The National Center for Biotechnology Information; the optimal annealing temperature was 60 °C. The primers were published in Schrickx [34].

## Data Availability

Data are contained within the article.
